# Predicting seasonal influenza epidemics using cross-hemisphere influenza surveillance data and local internet query data

**DOI:** 10.1038/s41598-019-39871-2

**Published:** 2019-03-01

**Authors:** Yuzhou Zhang, Laith Yakob, Michael B. Bonsall, Wenbiao Hu

**Affiliations:** 1 School of Public Health and Social Work; Institute of Health and Biomedical Innovation, Queensland University of Technology, Brisbane, Queensland Australia; 2 Faculty of Infectious and Tropical Diseases, London School of Hygiene & Tropical Medicine, London, UK; 3 Mathematical Ecology Research Group, Department of Zoology, University of Oxford, Oxford, UK

## Abstract

Can early warning systems be developed to predict influenza epidemics? Using Australian influenza surveillance and local internet search query data, this study investigated whether seasonal influenza epidemics in China, the US and the UK can be predicted using empirical time series analysis. Weekly national number of respiratory cases positive for influenza virus infection that were reported to the FluNet surveillance system in Australia, China, the US and the UK were obtained from World Health Organization FluNet surveillance between week 1, 2010, and week 9, 2018. We collected combined search query data for the US and the UK from Google Trends, and for China from Baidu Index. A multivariate seasonal autoregressive integrated moving average model was developed to track influenza epidemics using Australian influenza and local search data. Parameter estimates for this model were generally consistent with the observed values. The inclusion of search metrics improved the performance of the model with high correlation coefficients (China = 0.96, the US = 0.97, the UK = 0.96, p < 0.01) and low Maximum Absolute Percent Error (MAPE) values (China = 16.76, the US = 96.97, the UK = 125.42). This study demonstrates the feasibility of combining (Australia) influenza and local search query data to predict influenza epidemics a different (northern hemisphere) scales.

## Introduction

Determining the key drivers of the dynamics of seasonal and non-seasonal influenza outbreaks remains a major challenge^1^. Influenza epidemics typically occur during the winter months, which is considered May to October in the southern hemisphere and October through May in the northern hemisphere^2^. Influenza transmission alternates between the northern and southern hemispheres through these seasons. For instance, Southeast Asia was shown to maintain continuous circulation of influenza strain A (H3N2) which seeded transmission in Oceania and subsequently to North America, Europe and South America^3^. We hypothesize that influenza data from Australia is predictive of influenza transmission in the northern hemisphere’s impending seasonal influenza. Thus, status in Oceania countries may provide early warning for other countries, especially for countries in the northern hemisphere.

Through 2017, Australia experienced the severest influenza outbreak for five years with the predominant circulating influenza virus being H3N2^4^. Deaths reported in notified laboratory cases confirmed influenza was higher in 2017 (n = 745) than in recent years (5 year average = 176; Range: 28–745)^4^. This strain (in conjunction with influenza B virus) led to the largest influenza epidemic outbreak in the last five years in the northern hemisphere during the 2017–2018 season, up to 15th March, 2018, a total of 128 influenza-associated pediatric deaths and 327 confirmed deaths had been reported in the US and the UK respectively^5,6^.

There is often a delay of up to two weeks between the onset of influenza disease and when notification data is compiled into traditional surveillance reports^7^. This lag in reporting limits the ability for conventional surveillance systems to provide timely epidemiological intelligence and delays the response of health officers to mitigate or manage possible outbreaks^8^. Recently, there has been growing interest in using internet search metrics to perform rapid detection and surveillance for infectious diseases outbreaks^9–12^. This new tool relies on the premise that disease activity can be predicted by tracking changes in frequencies of related internet searches for key terms, as people will actively seek related information from the internet when they contract a disease in the early phase^13^. Although burgeoning, maximising the benefit of this new approach is still work in progress, with previous internet search data-based models sometimes producing erroneous projections^14–16^.

This study aims to develop an empirical time series model using Australian influenza surveillance and local internet search query data to predict seasonal influenza epidemics in the northern hemisphere. We will assess predictive performance separately for China, the US and the UK (these three countries account for 23.7% of the world population at the end of 2017^17^). Our aim is to improve upon predictive tools utilising only infection surveillance data through complementation with internet search data.

## Results

### Descriptive analysis of influenza epidemics

**Figure 1:**
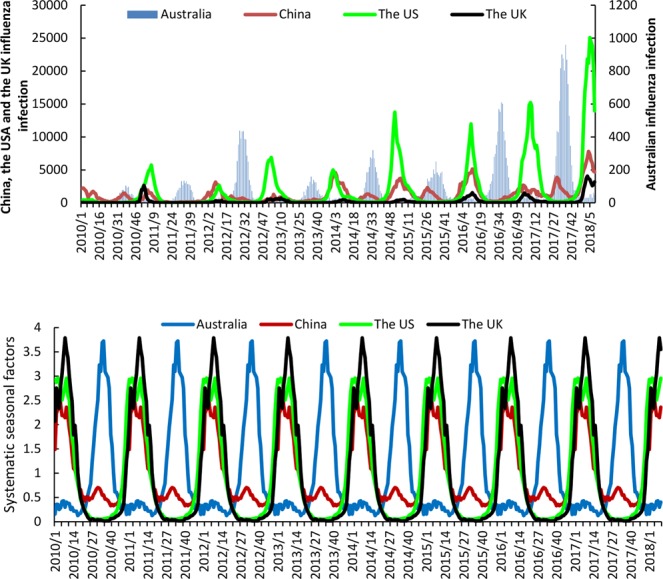
The trends (upper panel) and systematic seasonal factors (lower panel) of positive influenza virological number of Australia, China, the US and the UK between week 1 2010 and week 9 2018. (X axis: date (week), Systematic seasonal factors were generated by seasonal decomposition procedures).

The trends and systemic seasonal variation of influenza infection are illustrated in Fig. 1. From seasonal decomposition and time series analysis, clear seasonal patterns are evident for Australia, China, the US and the UK. The influenza epidemics were observed to peak between January and March in the US, the UK and China and between August and October in Australia. Moreover, different influenza subtypes predominated each year. However, for most of the study period, the strains circulating in the subsequent northern hemisphere influenza seasons of China, US and the UK were the same as those circulating in Australia (Supplementary Table 1)^18^.

### Time-series cross correlation analysis

**Figure 2:**
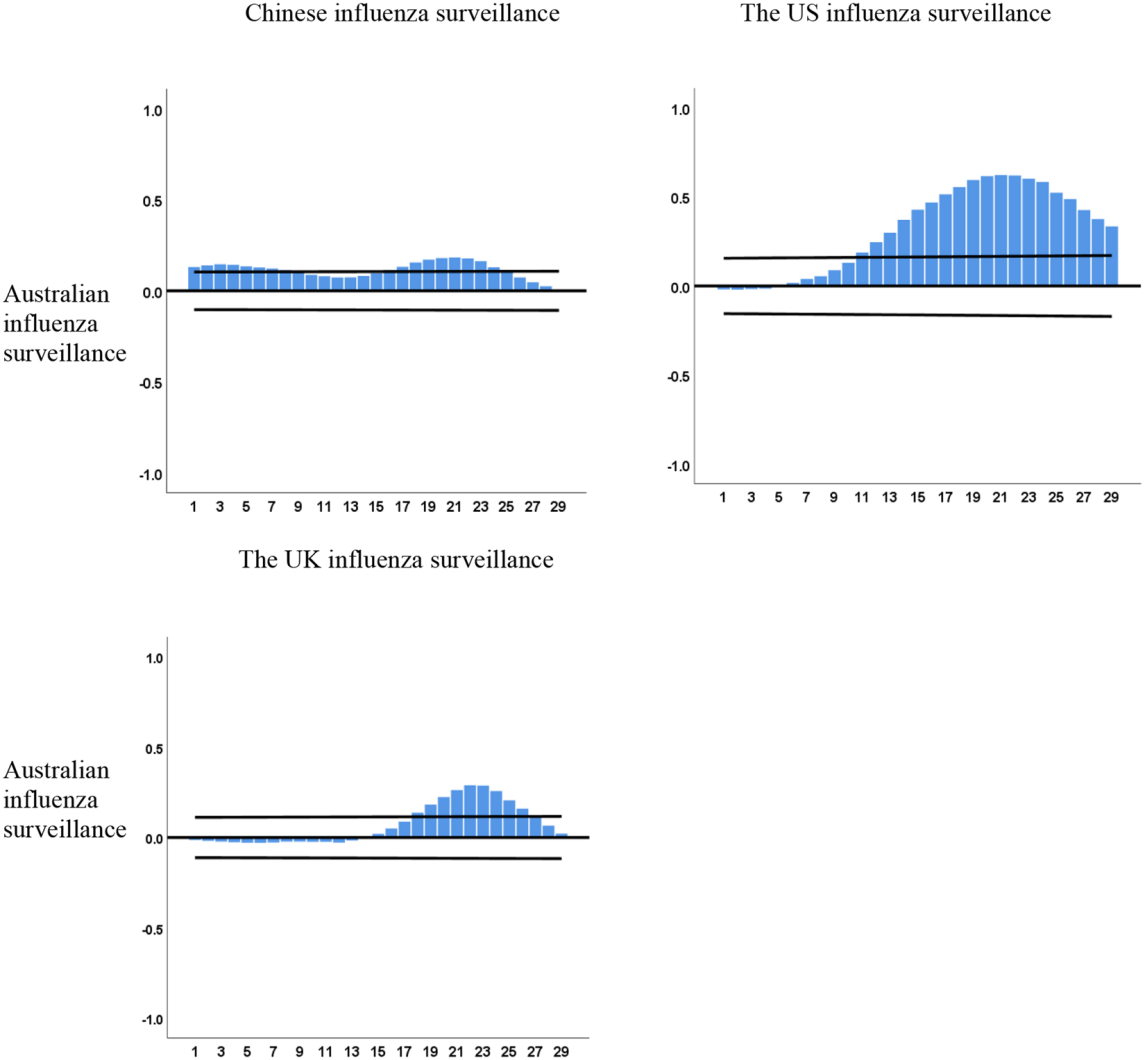
Cross-correlation between Australian influenza surveillance with Chinese, the US and the UK influenza surveillance data. Confidence intervals (95%) are indicated by the black lines (X axis: lag value, Y axis: CCF value).

The cross-correlation function (CCF) showed the strongest correlations between Australia with China, the US and the UK in the trends of influenza surveillance at a lag of 21 weeks (r = 0.184), 22 weeks (r = 0.622) and 22 weeks (r = 0.289) (all p < 0.05), respectively (Fig. 2) (Supplementary Table 2). Additionally, the CCFs showed that BI at a lag of 1 week had a significant association with seasonal influenza at China (r = 0.467); similarly, a 1-week lag had the closest association between GT and the US (r = 0.632) and the UK (r = 0.419) (all p < 0.05), separately (Supplementary Fig. 1) (Supplementary Table 3).

### Seasonal autoregressive integrated moving average (SARIMA) model

**Figure 3:**
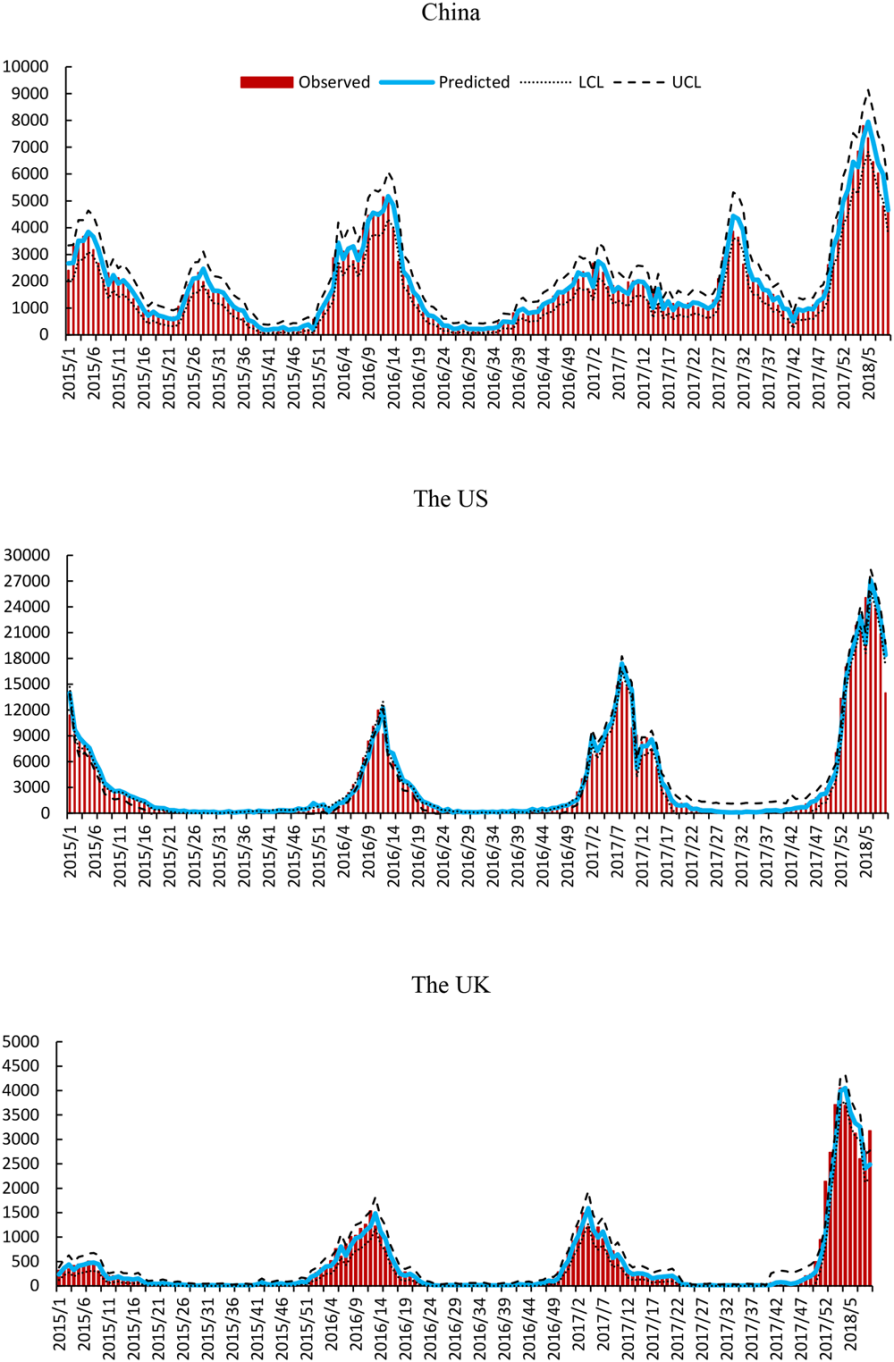
Weekly observed and 1-week ahead predicted positive influenza virological number using SARIMA model in China, the US and the UK from week 1, 2015 to week 9, 2018 (X axis: date (week), Y axis: positive influenza virological number, LCL: the lower control limit, UCL: the upper control limit).

Australian influenza data and local search query with maximal correlation coefficient were used to construct the time series models. Thus, we used Australian influenza at 21-week, 22-week and 22-week lag to construct the models for China, the US and the UK, respectively. The local search data at 1-week lag was also included in the model construction as an independent covariate. The SARIMA model (1,1,1) (1,0,2), (2,2,2) (2,0,0) and (3,0,2) (1,0,0) with Australian influenza and local search data was found to provide the best fit to the data in China, the US and the UK, respectively. The analysis of goodness-of-fit revealed that the SARIMA models fitted the data well, as the autocorrelation function (ACF) and partial autocorrelation (PACF) of the residuals of the models fluctuate around zero (Supplementary Fig. 2). The results reveal that the models including Australian influenza surveillance data and local internet query data fitted the reported influenza data better with larger R² and smaller BIC and RMSE values (Table 1). Thus, the models that incorporated lagged Australian influenza and lagged local search data were selected as the predictive models for validation. Results of SARIMA models are presented in Table 2. Fitted and 1-week ahead predicted values of SARIMA models are shown in Fig. 3 and Supplementary Fig. 3.

**Table 1:** The goodness-of –fit results of SARIMA models.

	China (1,1,1) (1,0,2)			US (2,2,2) (2,0,0)			UK (3,0,2) (1,0,0)		
	R^2^	BIC	RMSE	R^2^	BIC	RMSE	R^2^	BIC	RMSE
Model 1	92.80	13.17	301.55	94.20	14.33	557.46	91.60	12.27	139.23
Model 2	94.10	11.42	292.37	96.70	12.62	530.75	93.30	9.73	124.99
Model 3	93.90	11.87	297.41	96.40	12.94	536.18	92.90	10.06	128.37
Model 4	94.40	11.12	245.18	96.80	12.16	405.13	93.90	9.52	111.95

Model 1: Australian influenza data and local search data excluded model; Model 2: Australian influenza data included model; Model 3: Local search data included model; Model 4: Australian influenza data and local search data included model.

**Table 2:** Parameters estimates (and significance testing) associated with the SARIMA models for China, the US and the UK.

	Parameters	Coefficients	Standard error	t	P value
China	AR	0.75	0.10	7.68	0.000
	MA	0.52	0.13	4.12	0.000
	SAR	0.71	0.32	2.21	0.028
	SMA	0.73	0.33	2.21	0.028
	Search	0.16	0.04	4.57	0.001
	Influenza	0.01	0.01	2.17	0.031
The US	AR	1.65	0.04	40.91	0.000
	MA	1.99	0.02	81.24	0.000
	SAR	0.04	0.07	0.52	0.602
	Search	3.24	0.38	8.44	0.000
	Influenza	0.22	0.07	3.12	0.002
The UK	AR	0.95	0.02	54.60	0.000
	MA	0.85	0.08	6.13	0.000
	SAR	0.27	0.06	4.57	0.001
	Search	0.10	0.02	8.47	0.000
	Influenza	0.01	0.01	2.33	0.020

AR: autoregressive, MA: moving average, SAR: seasonal autoregressive, SMA: seasonal moving average, Search: local internet search metrics, Influenza: Australian influenza infection.

The evaluation of forecasting performance of the 1-week ahead predictive models is presented in Supplementary Table 4. This table shows that the predictive capacity of the models including Australian influenza surveillance data and local search data is higher with larger overall Pearson correlations (China = 0.96, the US = 0.97, the UK = 0.96, p < 0.01). The models were also robust as indicated by the smaller size of the overall MAPE (China = 16.76, the US = 96.97, the UK = 125.42), which measures the discrepancies between the off-target model predictions and the observed influenza notifications.

## Discussion

Here, we have used time series analysis and local internet search data to predict emerging influenza dynamics. To the best of our knowledge, this is the first attempt to combine Australian influenza surveillance and local internet search data to predict influenza epidemics in the northern hemisphere. We used FluNet, the positive virological data of variable flu strains to predict influenza epidemics in the study. Across years in which both the same strains dominated in the different hemispheres, our models demonstrated a good predictive capacity using Australia influenza surveillance data. Intrinsic strain virulence is only part of what makes up the force of infection, with other components including abiotic factors and population-level susceptibility^19^. We developed an early warning model combining the surveillance data from different geographic scales as the dramatic change in patterns of population movement over last decade has been reported as an important contributor for the transmission of infectious diseases^20^.

Seasonal time series decomposition analysis showed clear seasonal patterns of influenza infections in each country. Larger numbers of individuals reported with positive influenza viral loads were observed during winter and spring months across the eight-year study period. The strong association between the influenza surveillance data of China, the US and the UK with that of Australia during the previous influenza seasons indicated the significant potential for monitoring influenza epidemics in northern hemisphere using southern hemisphere’s influenza surveillance coupled with significant improved refinement, albeit at reduced lead-in time, when also combined with local internet search data.

Results of cross correlation showed that significant associations were exhibited at 1–7 week lag between local internet search data and influenza surveillance data. The signalling of variation in search metrics may offer sufficient time to implement influenza preventive measures. These results suggest that a pre-requisite for constructing early warning systems for influenza would be to combine southern hemisphere influenza data with local internet search data. The southern hemisphere data provides a multi-month lead-time while the internet query data significantly improves predictive performance just prior to the increased transmission in the northern hemisphere.

Our results indicate that time series models that combine opposite-hemisphere influenza surveillance data with internet search covariates allow a marginal increase in forecasting accuracy at one week ahead, as internet search data can be collated faster with emerging northern hemisphere epidemiological influenza trends. Several recent studies have demonstrated similarly improved forecasting capabilities when combining local surveillance data with internet data^21,22^. Linking variation in influenza infections of southern hemisphere with local internet search data could provide longer time windows for government and health authorities in northern hemisphere countries to implement cost-effective influenza preventive measures.

There is an opportunity for using search data in diseases surveillance in the countries (with strong information communications technology capacity) for monitoring emerging infectious diseases in vulnerable regions or where access to traditional disease surveillance is limited^23^. Moreover, collating these data can capture anomalous patterns of diseases in real-time, which is unlikely to be achieved by traditional surveillance^24^. The results of this study suggests the SARIMA models that included internet search query improve the ability to predict patterns in the time series.

It is important to bear in mind that there are some limitations with these sorts of studies that will only improve with better reporting structures. For instance, the FluNet only includes reported virological data from participating laboratory, and this database does not include patients who have influenza but do not seek medical care. Furthermore, it is acknowledged that different internet-seeking behaviours, self-reporting and media-driven biases exist between different sectors^11^: for example, previous studies reported that media bias can adversely influence internet-based surveillance systems^10,25^. These sources of (observation) error have obvious implications for developing precise predictions about epidemic outbreaks. Developing methods to deal with better reporting mechanisms and/or novel, contemporary statistical approaches should be future goals. Moreover, we used the total number of the positive virological data of variable flu strains in the study. Further work is needed to assess predictive performance when multiple flu strains are co-circulating at high levels.

All these issues notwithstanding, shifting patterns of health-seeking behaviour, the digitisation of society and increased internet access provide a unique opportunity to address emerging infectious disease events^9^. The internet provides online, real-time health-related data with high geographical resolution that can be systematically queried, aggregated and analysed to inform public health agencies^26^. The potential impact of disease outbreaks generally extends beyond the local scale. Surveillance combining traditional and internet data sources will have global relevance and could contribute to the improvement of global health security^9^. In the future, a dynamic and integrated spatiotemporal influenza early warning system, which incorporates uncertainty around the dynamic and heterogeneous asymptomatic rate of disease^27^ and that is developed by combining web search engine query data with socio-environmental factors and historic disease surveillance data may have the potential to assist public health authorities in identifying high risk areas on a global scale.

## Methods

### Data collection

Weekly data on national total number of individuals with influenza positive viruses that were reported to the FluNet surveillance system in Australia, China, the US and the UK were obtained from World Health Organization FluNet surveillance between week 1, 2010, and week 9, 2018^18^. FluNet is a global web-based tool for influenza virological surveillance, which was provided remotely by National Influenza Centres (NICs) of the Global Influenza Surveillance and Response System (GISRS). We used Google Trends (GT; for the US and the UK) and the Baidu Index (BI; for China) to collect search data. We selected ten top search terms, (the selected search terms for data analysis are showed in Supplementary Table 5) which highly correlated with the term “influenza” from Google Correlate (GC) for the US and the UK, and from BI for China, then we combined the selected search terms as one search query to collect search data. FluNet has 53 weeks’ surveillance data in 2015 and the number of influenza positive viruses in the week is relatively small. Thus, we aggregate the data of week 53, 2015 into week 52, 2015 in the study. Moreover, no overlap between the last week and first week of the following year was found in the weekly influenza surveillance data and search data.

### Data analysis

#### Descriptive analysis of influenza epidemics

All data analyses were performed with SPSS Statistics software, version 25 (SPSS Inc; Chicago, IL, USA). Statistical significance was set at P < 0.05. All data were checked for completeness and accuracy before analysis.

Time series decomposition procedures are able to describe the trend and seasonal factors in a time series^28^. The goal of this analysis was to determine systematic seasonal variations in influenza epidemics in the study period.

#### Time-series cross correlation analysis

To assess the correlations between Australian influenza infections and other countries’ data (the US, the UK and China), as well as the associations between the influenza epidemics data (the US, the UK and China) and their local search data, time-series cross correlation was undertaken. We used seasonal adjusted data in this analysis by performing seasonal decomposition analysis. As the variables are strongly associated with each other with different time lags, only those with maximal correlation coefficient were used to construct the models in the analysis^12,29^.

#### SARIMA model with Australian influenza and local search query data

As influenza has a strong seasonal characteristics in time series^30^, SARIMA models were developed to control the effects of seasonality in the forecast of influenza epidemics^10,31^. We used Australian influenza data and local search query as the independent variable to predict influenza epidemics of the US, the UK and China, separately. Generally, there are three significant components of a SARIMA model, an autoregressive (AR) component, a differencing and moving average (MA) component. These parameters are typically selected when fitting these model: (p, d, q) (P, D, Q); where p is the order of the AR, d is the order of the differencing, q is the order of the MA, P is the order of the seasonal AR, D is the order of the seasonal differencing, and Q is the order of the seasonal MA^32^.

The equation of this model is formulated as:

$${y}_{t}={{\rm{\Theta }}}_{q}(A){{\rm{\Theta }}}_{Q}({A}^{s}){a}_{t}{{\rm{\Phi }}}_{P}({A}^{s})/{{\rm{\Phi }}}_{P}({A}^{s}){{\rm{\Phi }}}_{p}(A){({1}-A)}^{d}{({1}-{A}^{s})}^{D}+AusInf+LocSch$$

where *Φ*_*P*_*(A*^*s*^) is seasonal autoregressive operator, *Φ*_*p*_*(A*) is autoregressive operator, *Θ*_*q*_*(A)* is the operator of moving averages, *Θ*_*Q*_*(A*^*s*^*)* is seasonal operator of moving averages, *a*_*t*_ is white noise, *y*_*t*_ is predicted influenza surveillance data, *AusInf* and *LocSch* are Australian influenza surveillance data and local search data’s regressive coefficients.

To test the goodness-of-fit of the model for training period, autocorrelation and partial autocorrelation of residuals were assessed. In addition, Bayesian information criterion (BIC), the stationary R square (R²) and the Root Mean Squared Error (RMSE) were also used to examine the goodness-of-fit of the model in training period. The data file was divided into two data sets: data from week1, 2010 to week 52, 2014 was used as a training dataset to construct models and data from week 1, 2015 to week 9, 2018 was used as a test data set to validate the models. Additionally, a comparison of performance of SARIMA models that either included or excluded local search data was undertaken. A particular SARIMA model can be considered an improvement relative to others if it has a large R² value and a small BIC value. The better fitting model was selected for use as the predictive model. Here, we reported three metrics to evaluate the predictive performance of the SARIMA model in validation period: Pearson correlation, RMSE and the Maximum Absolute Percent Error (MAPE)^12,33^.

## Supplementary information

Supplementary information

## Data Availability

The datasets analysed during the current study are calculated based on the methods described in this study and the original data of the influenza surveillance, which can be found at www.who.int/influenza/gisrs_laboratory/flunet/en/.
