# Supplementary material for: Predicting seasonal influenza epidemics using cross-hemisphere influenza surveillance data and local internet query data
Source: Sci Rep. 2019 Mar 1;9:3262. doi: 10.1038/s41598-019-39871-2 (PMC6397245; doi:10.1038/s41598-019-39871-2)
Supplement: Supplementary file 1 — Supplementary information [file 41598_2019_39871_MOESM1_ESM.pdf]

Supplementary information

Predicting seasonal influenza epidemics using cross-hemisphere influenza surveillance data and local internet query data

AUTHORS

Yuzhou Zhang [1], Laith Yakob [2], Michael B. Bonsall [3] and Wenbiao Hu [1]*

AUTHOR AFFILIATIONS

1. School of Public Health and Social Work; Institute of Health and Biomedical Innovation, Queensland University of Technology, Brisbane, Queensland, Australia. (Yuzhou Zhang: yuzhou.zhang@hdr.qut.edu.au; Wenbiao Hu: w2.hu@qut.edu.au);
2. Faculty of Infectious and Tropical Diseases, London School of Hygiene & Tropical Medicine, London, UK. (Laith Yakob: Laith.Yakob@lshtm.ac.uk);
3. Mathematical Ecology Research Group, Department of Zoology, University of Oxford, Oxford, UK. (Michael B. Bonsall: michael.bonsall@zoo.ox.ac.uk)

CORRESPONDING AUTHOR

Wenbiao Hu, School of Public Health and Social Work; Institute of Health and Biomedical Innovation, Queensland University of Technology, Brisbane, Queensland, Australia. Phone: +61 31385724 Email: w2.hu@qut.edu.au;

Figure S1. Cross-correlation between Chinese, American and British influenza surveillance data and local search data. Confidence intervals (95%) are indicated by the black lines (X axis: lag value, Y axis: CCF value).

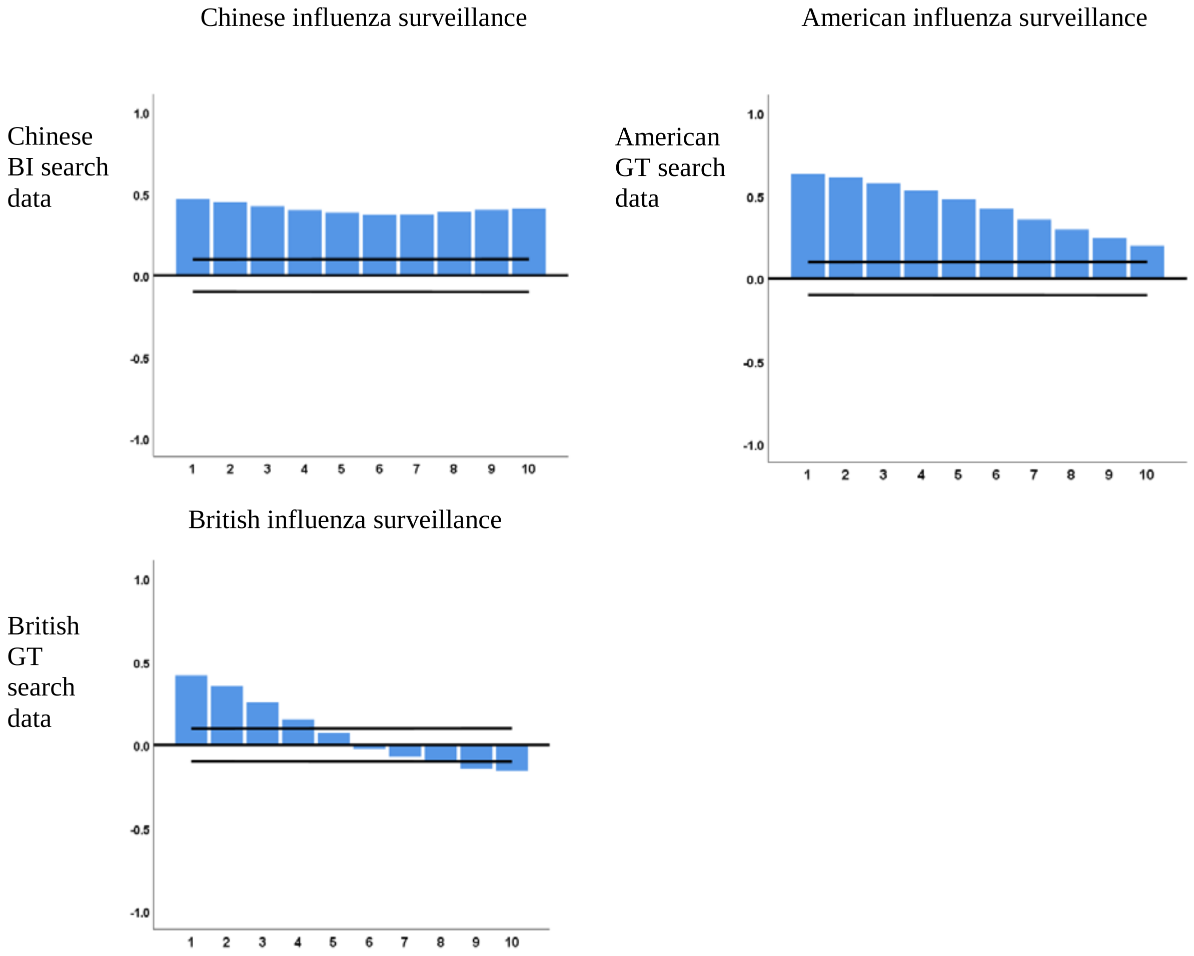

Figure S2. Autocorrelation function (ACF) and partial autocorrelation function (PACF) of the residual series of the SARIMA models.

The SARIMA model (1,1,1) (1,0,2) of China

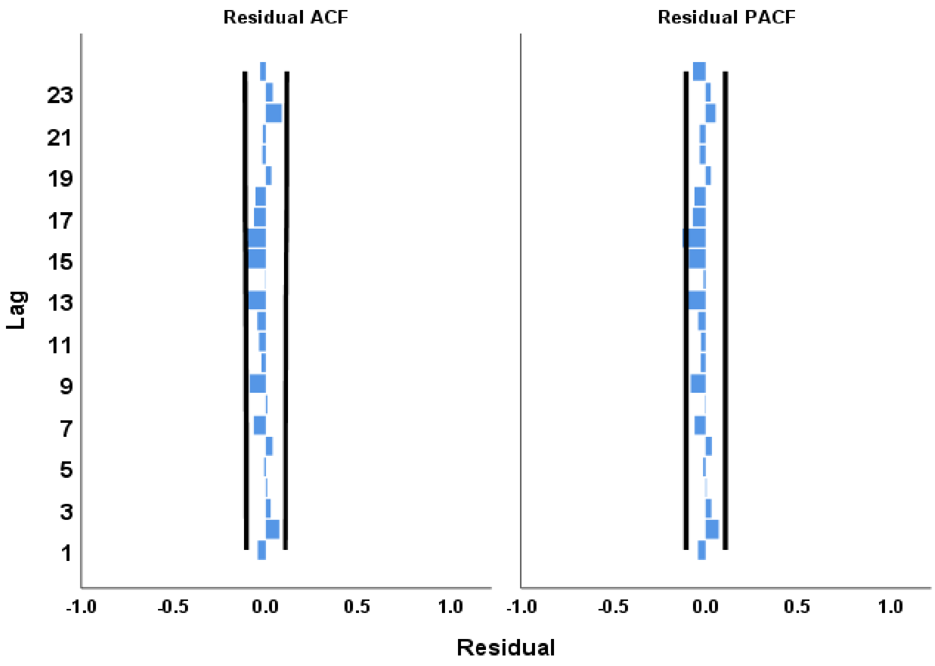

The SARIMA model (2,2,2) (2,0,0) of the US

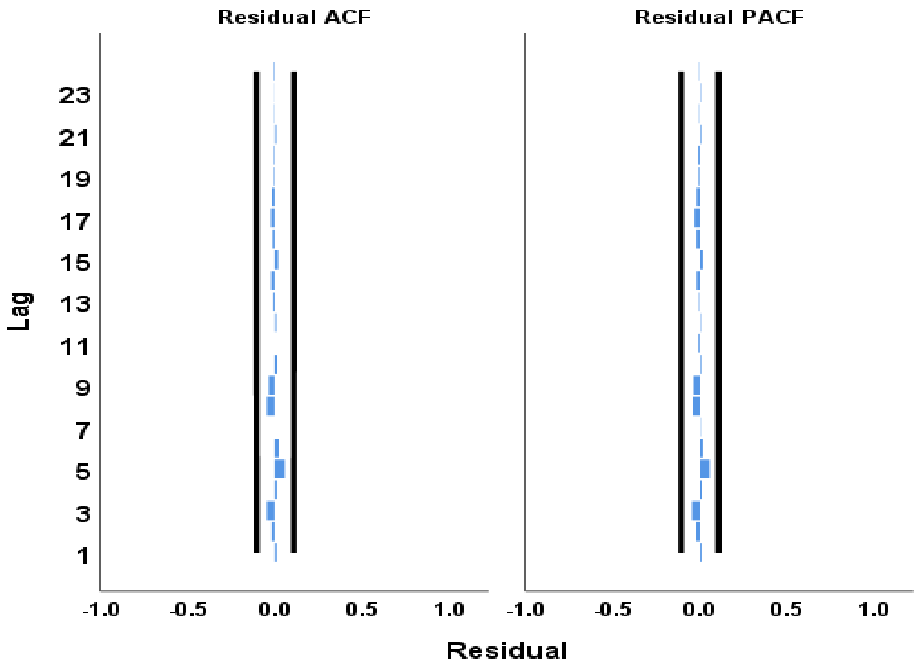

The SARIMA model (3,0,2) (1,0,0) of the UK

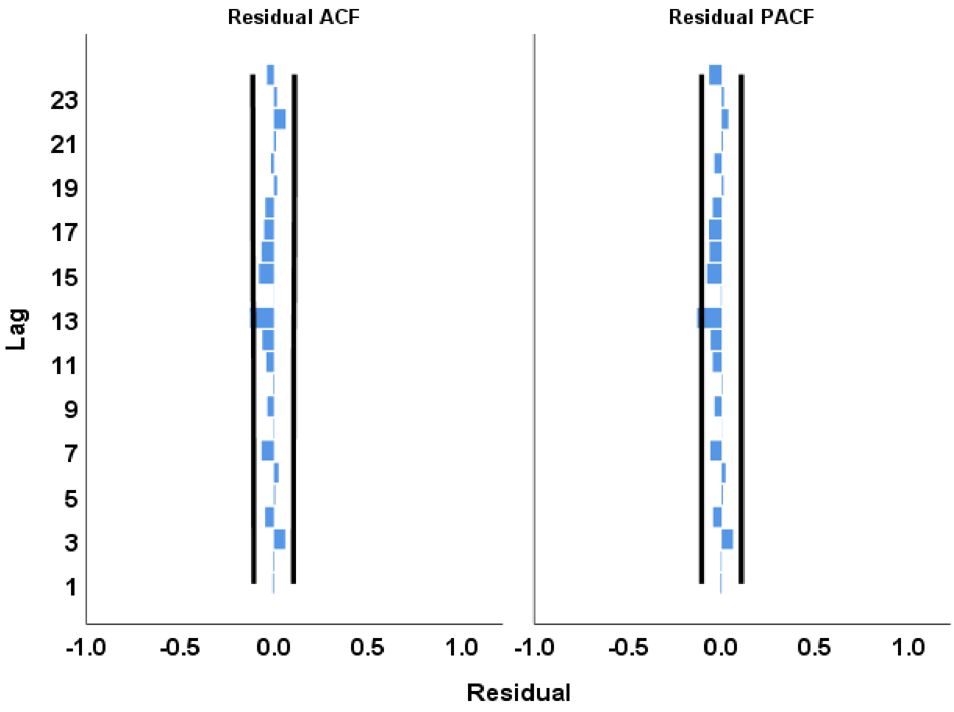

Figure S3. Weekly observed and fitted influenza infection using 1-week ahead SARIMA model in China, the US and the UK (LCL: the lower control limit, UCL: the upper control limit).

The SARIMA model (1,1,1) (1,0,2) of China

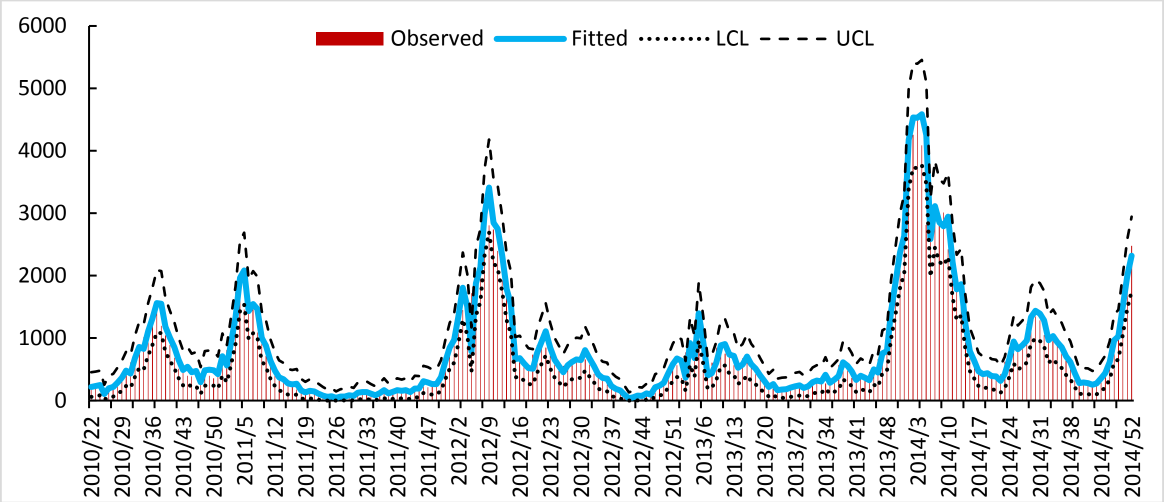

The SARIMA model (2,2,2) (2,0,0) of the US

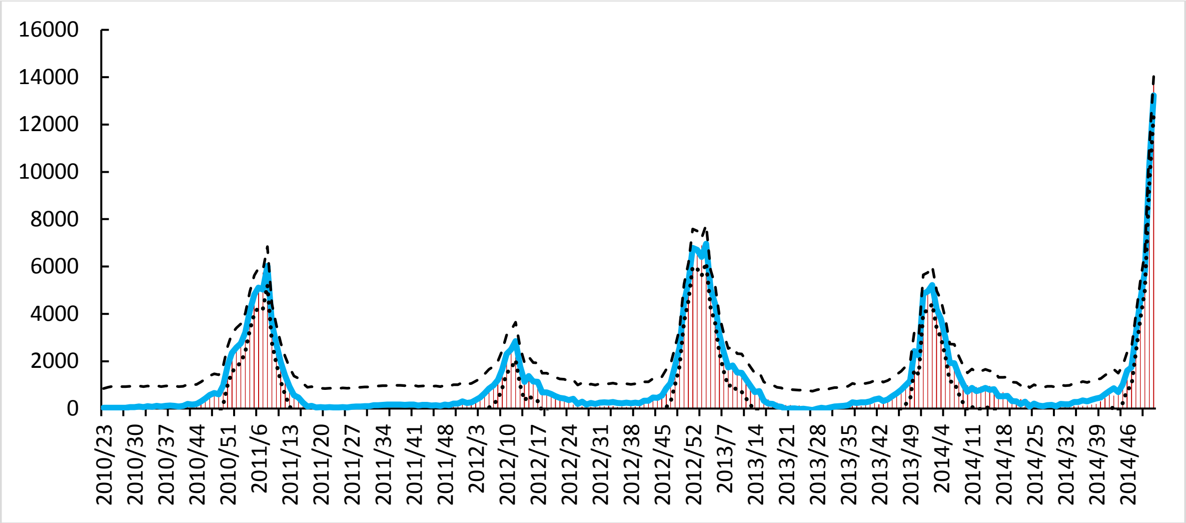

The SARIMA model (3,0,2) (1,0,0) of the UK

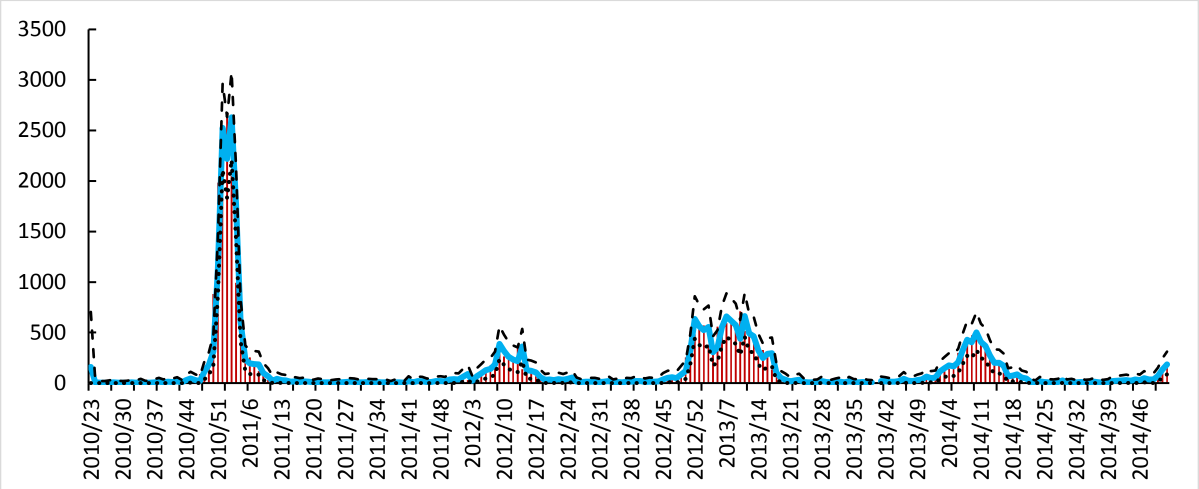

Table S1. The reported predominating influenza strains in influenza surveillance of Australia, China, the US and the UK from 2010 to 2017. (The surveillance data were the subsequent influenza season for China, the US and the UK relative to Australia)

Year	2010	2011	2012	2013	2014	2015	2016	2017
**Australia**	H3N2	H1N1	H3N2	H1N1	H1N1	H1N1	H3N2	H3N2
**China**	H3N2	H3N2	H3N2	H1N1	H3N2	H1N1	H3N2	H1N1
**The US**	H3N2	H3N2	H3N2	H1N1	H3N2	H1N1	H3N2	H3N2
**The UK**	H3N2	H2N3	H3N2	H1N1	H3N2	H1N1	H3N2	H3N2

Table S2. Cross-correlation between Australian influenza surveillance with Chinese, the US and the UK influenza surveillance data.

	China		The US		The UK	
**Lag (weeks)**	**Correlation**	**Std. Error**	**Correlation**	**Std. Error**	**Correlation**	**Std. Error**
1	0.131	0.05	-0.019	0.05	-0.014	0.05
2	0.140	0.05	-0.021	0.05	-0.018	0.05
3	0.146	0.05	-0.017	0.05	-0.023	0.05
4	0.143	0.05	-0.015	0.05	-0.026	0.05
5	0.134	0.05	0.002	0.05	-0.028	0.05
6	0.128	0.05	0.017	0.05	-0.029	0.05
7	0.122	0.05	0.040	0.05	-0.027	0.05
8	0.113	0.05	0.055	0.05	-0.023	0.05
9	0.101	0.05	0.088	0.05	-0.023	0.05
10	0.088	0.05	0.130	0.05	-0.024	0.05
11	0.080	0.05	0.188	0.05	-0.024	0.05
12	0.073	0.05	0.246	0.05	-0.028	0.05
13	0.074	0.05	0.299	0.05	-0.016	0.05
14	0.082	0.05	0.371	0.05	-0.001	0.05
15	0.096	0.05	0.428	0.05	0.020	0.05
16	0.112	0.05	0.468	0.05	0.050	0.05
17	0.132	0.05	0.515	0.05	0.088	0.05
18	0.156	0.05	0.555	0.05	0.137	0.05
19	0.171	0.05	0.595	0.05	0.182	0.05
20	0.180	0.05	0.617	0.05	0.224	0.05
21	0.184	0.05	0.620	0.05	0.262	0.05
22	0.178	0.05	0.622	0.05	0.289	0.05
23	0.163	0.05	0.603	0.05	0.287	0.05
24	0.130	0.05	0.584	0.05	0.257	0.05
25	0.104	0.05	0.523	0.05	0.206	0.05
26	0.074	0.05	0.488	0.05	0.160	0.05
27	0.047	0.05	0.426	0.05	0.119	0.05
28	0.025	0.05	0.375	0.05	0.066	0.05
29	0.002	0.05	0.335	0.05	0.021	0.05

Table S3. Cross-correlation between Chinese, the US and the UK influenza surveillance data and local search data.

	China		The US		The UK	
**Lag (weeks)**	**Correlation**	**Std. Error**	**Correlation**	**Std. Error**	**Correlation**	**Std. Error**
1	0.467	0.05	0.632	0.05	0.419	0.05
2	0.449	0.05	0.612	0.05	0.355	0.05
3	0.425	0.05	0.577	0.05	0.257	0.05
4	0.401	0.05	0.532	0.05	0.156	0.05
5	0.387	0.05	0.480	0.05	0.075	0.05
6	0.373	0.05	0.424	0.05	-0.024	0.05
7	0.375	0.05	0.358	0.05	-0.071	0.05
8	0.390	0.05	0.296	0.05	-0.101	0.05
9	0.402	0.05	0.245	0.05	-0.144	0.05
10	0.411	0.05	0.200	0.05	-0.154	0.05

Table S4. Similarity metrics between observed influenza surveillance and fitted value using SARIMA model (Model 4: Australian influenza data and local search data included model; Model 1: Australian influenza data and local search data excluded model; data period: week 1 2010 to week 9, 2018).

	China			US			UK		
	**Correlation**	**RMSE**	**MAPE**	**Correlation**	**RMSE**	**MAPE**	**Correlation**	**RMSE**	**MAPE**
**2010 (Model 4)**	0.94*	89.34	11.49	0.93*	95.84	301.58	0.98*	19.51	144.66
**(Model 1)**	0.91*	91.56	12.32	0.90*	96.71	303.26	0.96*	20.63	147.17
**2011 (Model 4)**	0.96*	44.10	21.05	0.97*	90.30	286.79	0.99*	14.23	189.90
**(Model 1)**	0.93*	47.83	22.57	0.95*	91.85	288.41	0.97*	15.75	190.41
**2012 (Model 4)**	0.95*	78.41	19.96	0.99*	225.24	33.25	0.88*	43.97	74.24
**(Model 1)**	0.94*	80.62	21.09	0.97*	227.36	35.17	0.85*	45.22	75.53
**2013 (Model 4)**	0.94*	157.74	21.51	0.98*	244.24	27.48	0.96*	19.20	138.97
**(Model 1)**	0.91*	159.15	23.26	0.96*	247.18	28.83	0.95*	21.56	140.69
**2014 (Model 4)**	0.97*	110.16	12.99	0.98*	561.09	29.32	0.98*	12.15	125.69
**(Model 1)**	0.94*	112.37	14.43	0.96*	562.27	31.29	0.97*	15.08	126.80
**2015 (Model 4)**	0.97*	118.13	16.84	0.99*	137.41	43.52	0.97*	18.26	85.48
**(Model 1)**	0.96*	121.28	18.37	0.98*	139.09	44.65	0.95*	20.07	87.34
**2016 (Model 4)**	0.98*	130.48	13.32	0.97*	321.25	31.39	0.97*	48.89	60.97
**(Model 1)**	0.96*	131.51	14.86	0.95*	323.77	33.34	0.96*	51.34	62.64
**2017 (Model 4)**	0.97*	253.31	16.91	0.98*	798.36	22.45	0.97*	168.79	183.41
**(Model 1)**	0.95*	256.32	18.52	0.96*	801.81	24.06	0.94*	170.90	185.12
**Overall (Model 4)**	0.96*	122.71	16.76	0.97*	309.22	96.97	0.96*	43.13	125.42
**(Model 1)**	0.94*	125.08	18.18	0.95*	311.26	98.63	0.94*	45.07	126.96

*: P<0.01.

Table S5. The search queries used in data analysis

China	The US	The UK
Influenza	Influenza	Influenza
Avian flu	Influenza virus	Flu
Influenza virus	Flu	Influenza virus
Influenza prevention	Influenza death	Influenza vaccine
Influenza B	Flu medication	Influenza symptoms
Influenza symptoms	Flu spread	Influenza UK
Influenza treatments	Flu signs	Influenza treatment
Influenza vaccine	Flu viruses	Avian influenza
Flu	Flu reports	Influenza medication
2018 influenza	Flu cure	Influenza B
